# Lacrimal gland injection of platelet rich plasma for treatment of severe dry eye: a comparative clinical study

**DOI:** 10.1186/s12886-022-02554-0

**Published:** 2022-08-13

**Authors:** Mai A. Mohammed, Ibrahim Y. Allam, Mohamed Shafik Shaheen, Sihem Lazreg, Mohamed Fahmy Doheim

**Affiliations:** 1 Ophthalmology Department, Faculty of Medicine, Alexandria University, Alexandria, Egypt; 2 Cabinet Ophtalmologie, Alger Centre, Algiers, Algeria

**Keywords:** Platelet rich plasma, Lacrimal gland injection, Dry eye, Sjogren syndrome

## Abstract

**Background:**

We aimed to assess the efficacy of the injections of platelet rich plasma (PRP) for the treatment of severe dry eye disease (DED).

**Results:**

In this retrospecitve interventional clinincal study, we included 28 eyes of 14 patients with severe DED who were diagnosed with Sjogren syndrome. Each patient received unilateral lacrimal gland injection of PRP at days 0, 30, 60 and 90 days while the other eye served as control group who received preservative free eye drops. We objectively assessed parameters at baseline, 1 month, 2 months and 3 months including ocular surface analyzer (OSA) namely; noninvasive tear breakup times (NIBUT), tear meniscus height (TMH), lipid layer thickness (LLT) in addition to the Schirmer test I, corneal fluorescein staining (CFS) and meiboscore. The mean age was 43.4 ± .7.85 years. Comparing different parameters, baseline data showed non-significant difference between injected eye group and control group. After 1 and 3 months of follow up, there were significant differences between both groups regarding NITBUT, TMH, LLT, CFS and Schirmer test, with *p* <  0.001 in favor of PRP group.

**Conclusion:**

Injection of PRP in lacrimal gland is simple, safe, and effective technique in treatment of severe dry eye; proved by improvement of tear film parameters through subjective and objective assessment. Further studies are needed to standardize the technique and to confirm these results.

## Introduction

The eye health counts on the tear film to provide the needed moisture and lubrication which maintain both vision and comfort. Dry eye disease (DED) is defined at TFOS/DEWS II as “a multifactorial disease of the ocular surface characterized by a loss of homeostasis of the tear film and accompanied by ocular symptoms, in which tear film instability and hyperosmolarity, ocular surface inflammation and damage, and neurosensory abnormalities play etiological roles” [1]. It is a common disorder and the incidence of DED increases with age with a higher prevalence in women compared to men [2]. Moreover, it is a disabling disorder affecting the quality of life, visual function and has a significant socioeconomic impact. Currently, there is no cure for DED and treatments are targeted to manage the symptoms namely artificial tears which give symptomatic relief by keeping the ocular surface moistened without resolving underlying causes. The use of human blood as a source of a wide range of cell-based or protein-based therapeutic products is considered as a key element in regenerative medicine and platelet derived preparations, rich in growth factors, are now increasingly used for several therapeutic applications including tissue repair, wound healing, and regeneration [3].

The use of blood derived products such as platelet rich plasma (PRP) and the autologous serum (AS) is regarded as a new treatment modality for severe dry eye as that can enrich the ocular surface with growth factors in addition to the anti-inflammatory cytokines. This can be explained by the fact that these blood derived products have equivalent components that healthy natural tears have such as vitamins, growth factors, nutrients, and fibronectin. Therefore, the use of AS as a tear substitute can be feasible [4]. Platelets are activated along AS production and the content of their granules (growth factors, cytokine, and other proteins) are released to the supernatant and used as eye drops for the treatment of different ocular surface disorders. One of the main differences between PRPs and AS is that platelets are not enriched during AS production in comparison to PRP where platelets are enriched at least two-fold the platelet concentration in peripheral blood [5]. PRP, defined as a “volume of plasma containing a platelet concentration over the basal level (150 000–350 000/μl)”, prepared after a single or double centrifugation of total plasma, may have more merits over the AS [6]. In addition, the PRP preparation can take full merits of the plasma, the concentrated platelets and the stored growth factors by eliminating pro-inflammatory agents released by the leucocytes [7]. Platelets are able to store a broad array of biologically active agents as granules into vesicles such as growth factors such as epidermal growth factor (EGF), transforming growth factor (TGF), fibroblast growth factor (FGF), platelet derived growth factor (PDGF), nerve growth factor (NGF), and insulin-like growth factor (IGF), and cytokines and chemokines [8]. The high concentration of growth factors, anti-inflammatory cytokines, and other platelet derivatives may be beneficial for restoring the ocular surface in moderate to severe DED [9, 10].

The tear components generated by the lacrimal gland (LG) are vital in several processes related to the health of ocular surface and diseases that influence the lacrimal gland can lead to deficiency of aqueous tear and alteration in the ocular surface homeostasis that may result from radiation, inflammation, aging, or infection [11–13]. Research work targets regenerating in situ lacrimal gland in order to stimulate lacrimal gland secretions. Very few studies used the PRP as lacrimal gland injection for treatment of DED [14, 15]. For instance, a case series of patients with severe DED in which PRP was injected directly into the lacrimal gland leaded to an increase in lacrimal production and improvement of the symptoms of dry eye [14]. The present study is a non-randomised interventional consecutive study that retrospectively evaluated the efficacy of lacrimal gland injection of autologous PRP as a treatment tool in severe forms of chronic DED.

## Methods

### Study design

In this retrospecitve clinical study, we included 28 eyes of 14 patients with severe DED due to Sjogren syndrome. The diagnosis of primary and secondary Sjögren’s syndrome were made by a specialized rheumatologist in accordance with the criteria proposed by the American-European Consensus Group which identifies six criteria: “(I) ocular symptoms (dry eye symptoms, foreign body sensation, use of artificial tears three or more times per day); (II) oral symptoms (dry mouth, swollen salivary glands, need for liquids to swallow dry foods); (III) ocular signs (Schirmer test < 5 mm/5′, positive ocular surface staining); (IV) histopathology of salivary glands positive for focal lymphocytic sialadenitis; (V) oral signs (unstimulated whole salivary flow ≤1.5 mL/15′, abnormal parotid sialography, abnormal salivary scintigraphy); (VI) positivity of autoantibodies Anti-SSA (Ro) or Anti-SSB (La). The diagnosis of SS is reached with any four of the six criteria, including either item IV or VI; or with any three of the four objective criteria (III, IV, V, VI)” [16]. All patients with SS were receiving therapy with hydroxychloroquine (200 mg, once a day).

The study adhered to the tenets of the declaration of Helsinki and Institutional Review Board (the ethical committee of Alexandria University Faculty of Medicine) approval was obtained. We included cases with ocular surface disease index (OSDI) score ≥ 33, chronic dry eye > 6 months, Schirmer’s test values of < 5 mm after 5 minutes, and fluorescein score ≥ 3. Patients with previous refractive surgery, previous cataract or refractive surgeries, active ocular surface disease except dry eye, blinking abnormalities, contact lens wearers, and previous herpetic keratitis were excluded. Each patient received unilateral lacrimal gland injection of PRP while the other eye served as control group. Both groups received preservative free eye drops as needed and stopped at the day of examination.

Following the OSDI questionnaire, noninvasive testing for tear meniscus height (TMH), non-invasive tears break up time (NITBUT), lipid layer thickness (LLT) and meibography parameters were done prior to invasive testing, including Shirmer test I and CFS.

### Study assessment and parameters

Complete ophthalmic examination in the form of best corrected visual acuity, ocular adnexa and anterior segment examination by slit lamp biomicroscopy were performed. Subjective questionnaire of all patients using the Ocular Surface Disease Index (OSDI) were performed. An OSDI score of 12 was considered to be an indication of a normal healthy eye, a score of 13–22 was considered to be an indication of a mild dry eye condition, 23–32 was considered to be a sign of moderate dye eye condition, and a score of more than 33 indicated severe eye dryness [17], only patients with OSDI > 33 were included in the study. We used ocular surface analyzer SBM Sistemi machine (LS-3, Sun Kingdom co.,ltd, China) for assessment of the average of noninvasive tear breakup times (NIBUT) in seconds, tear meniscus height (TMH) in millimeters, lipid layer thickness (LLT) in nanometers; and infrared Meibography for Meiboscore calculation where the meibomian gland loss area was assessed for partial or complete loss of the MGs and scored using the following grades for each eyelid: 0, no loss of MGs; 1, area loss less than one-third of the total MG area; 2, area loss between one-third and two-thirds; 3, area loss more than two-thirds [18]. Lacrimal volume was assessed using Schirmer I test without anesthesia; regular Schirmer stripes (IO Schirme Eye Care Products, Delhi), were folded and gently placed to the temporal angle as far as practicable over the lower lid edge. During the procedure, the patient was advised to hold the eyes closed. The strips were calculated using the millimeter scale of each strip after 5 min of wetting. Corneal fluorescein staining (CFS) was tested according to the area (A) and density (D) classification of corneal fluorescein staining where the AD classification was graded using the scale reported by Miyata et al. (2003) (A) – 0, no punctate staining; 1, staining involving less than one-third of the cornea; 2, staining involving one-third to two-thirds of the cornea; 3, staining involving more than two-thirds of the cornea. (D) – 0, No punctate staining; 1, sparse density; 2, moderate density; 3, high density and overlapping lesions [19].

### Platelet rich plasma preparation

The patient’s blood was extracted into 10-mL sterile tubes containing 1 mL 3.8% sodium citrate acting as an anticoagulant. Centrifugation of total blood at optimal condition was used to achieve enrichment of platelets in plasma fraction. A first centrifugation with low forces (10 min at soft spin from 300 g) separates the whole blood into three layers: an upper layer that contains mostly platelets and white blood cells (WBC) called platelet-poor plasma (PPP); an intermediate thin layer of whitish color called buffy coat (BC), rich in WBC; and a bottom layer that consists mostly of red blood cells (RBC). The upper layer and superficial buffy coat was transferred into another sterile tube for a second centrifugation step at higher speeds (10 min at hard spin from 1200 g) to concentrate platelets. The upper two-thirds of the volume (PPP) were discarded, while the lower one-third was homogenized by gently shaking the tube to create PRP. Then, 1 ml of pure PRP were collected and platelets were activated adding 10% calcium chloride (CaCl2) in a proportion 0.05:1 mL (CaCl2: PRP) just before application. 1 ml of PRP was injected transcutaneous into the external one-third of the orbital rim at a depth of 4 mm to the superior area [14, 15, 20]. Injections were performed by one surgeon (IYA) and the surgeon who performed the injection was not the same as the one who evaluated the patient. Postoperative care included observation of the area. There were no negative side effects, including erythema, edema or severe pain, and analgesics that may alter platelet function were avoided.

### Follow up

Both injected eye and control group were followed up at 1, 2, and 3 months after initiation of therapy for symptoms and ocular surface parameters changes. Our follow up measures included NIBUT, TMH, LLT, Meiboscore, Schirmer test I and CFS.

### Statistical analysis

Statistical analyses were performed by SPSS version 25 (Statistical Packages for the Social Sciences, Chicago, Illinois, USA). Qualitative data were described using number and percent. We described quantitative data using mean and standard deviation. The Kolmogorov-Smirnov test was used to assess the normality. To compare both groups and baseline data to each point of follow up in the injected eye group, either paired t-test or the Wilcoxon signed ranks test were carried out. *P*-value of < 0.05 was considered to be statistically significant.

## Results

### Baseline characteristics for the included patients

In the present study, patients with severe aqueous deficient dry eye with a mean age of 43.4 ± .7.85 years (range between 28 and 56 years) of which 3 males (21.4%) and 11 females (78.6%) were retrospectively evaluated for unilateral lacrimal gland injection of PRP, and the other eye served as control group. Comparing all parameters, baseline data showed non-significant difference between injected eye group and control group except for corneal fluorescein staining (*p* = 0.029) (Table 1).

**Table 1:** Comparison of different parameters between cases and controls at baseline

Parameter	Cases	Controls	*P*-value
Schirmer test I	5.14 ± 1.29	5.21 ± 1.12	0.869
TMH	0.12 ± 0.02	0.11 ± 0.01	0.069
NITBUT	7.86 ± 1.29	8.14 ± 1.09	0.263
LLT	64.43 ± 4.84	63.57 ± 3.34	0.579
CSF	3.21 ± 0.579	2.71 ± 0.469	0.029
Meiboscore			
Upper	2.93 ± 0.267	2.93 ± 0.616	> 0.999
Lower	3.14 ± 0.77	3.07 ± 0.829	0.828

*Abbreviations*: *NIBUT* Noninvasive tear breakup times, *TMH* Tear meniscus height, *LLT* Lipid layer thickness, *CSF* Corneal fluorescein staining

### Study parameters at one month

After 1 month of follow up, there were significant differences between both groups regarding N1TBUT, TMH, LLT, Schirmer test I, and CSF and with *p* <  0.001 in favor of the PRP group (Table 2). However, the difference was not statistically significant for meiboscore for upper and lower lids with *p* = 0.165 for each respectively.

**Table 2:** Comparison of different parameters between cases and controls after 1 month

Parameter	Cases	Controls	*P*-value
Schirmer test I	10.14 ± 1.61	6.86 ± 0.94	< 0.001
TMH	0.18 ± 0.02	0.14 ± 0.02	< 0.001
NITBUT	11.07 ± 0.99	8.43 ± 1.82	< 0.001
LLT	73.57 ± 5.93	63.07 ± 3.33	< 0.001
CSF	1.50 ± 0.76	2.43 ± 0.514	0.004
Meiboscore			
Upper	2.71 ± 0.611	2.43 ± 0.514	0.165
Lower	2.86 ± 0.535	3.14 ± 0.535	0.165

*Abbreviations*: *NIBUT* Noninvasive tear breakup times, *TMH* Tear meniscus height, *LLT* Lipid layer thickness, *CSF* Corneal fluorescein staining

### Study parameters at two months

The difference continued to show statistical significance after 2 months comparing both groups regarding NITBUT, TMH, LLT, CFS and Schimer’s with *p* <  0.001 (Table 3). However, the difference was not statistically significant for Meiboscore for upper and lower lids.

**Table 3:** Comparison of different parameters between cases and controls after 2 months

Parameter	Cases	Controls	*P*-value
Schirmer test I	11.71 ± 0.99	6.79 ± 0.8	< 0.001
TMH	0.2014 ± 0.01	0.1407 ± 0.01	< 0.001
NITBUT	11.93 ± 1.07	9.21 ± 1.42	< 0.001
LLT	77.79 ± 4.57	64.36 ± 3.24	< 0.001
CSF	1.5 ± 0.76	2.36 ± 0.497	0.001
Meiboscore			
Upper	2.57 ± 0.514	2.79 ± 0.426	0.336
Lower	2.93 ± 0.616	2.93 ± 0.73	> 0.999

*Abbreviations*: *NIBUT* Noninvasive tear breakup times, *TMH* Tear meniscus height, *LLT* Lipid layer thickness, *CSF* Corneal fluorescein staining

### Study parameters at three months

After 3 months, the difference persisted and showed to be statistically significant comparing both groups regarding, NITBUT, TMH, LLT, CSF and Schimer’s, with *p* <  0.001 (Table 4). However, the difference was not statistically significant for Meiboscore for upper and lower lids.

**Table 4:** Comparison of different parameters between cases and controls after 3 months

Parameter	Cases	Controls	*P*-value
Schirmer test I	12.64 ± 0.929	7.86 ± 1.351	< 0.001
TMH	0.21 ± 0.01038	0.1486 ± 0.02685	< 0.001
NITBUT	12.07 ± 0.997	9.57 ± 1.399	< 0.001
LLT	80.86 ± 3.820	67.14 ± 3.207	< 0.001
CSF	0. 86 ± 0.663	2.36 ± 0.497	< 0.001
Meiboscore			
Upper	2.57 ± 0.646	2.86 ± 0.663	0.302
Lower	3 ± 0.679	3.07 ± 0.829	0.793

*Abbreviations*: *NIBUT* Noninvasive tear breakup times, *TMH* Tear meniscus height, *LLT* Lipid layer thickness, *CSF* Corneal fluorescein staining

### Parameters at baseline compared to 1, 2, and 3 months in the injected eye group

**Fig. 1:**
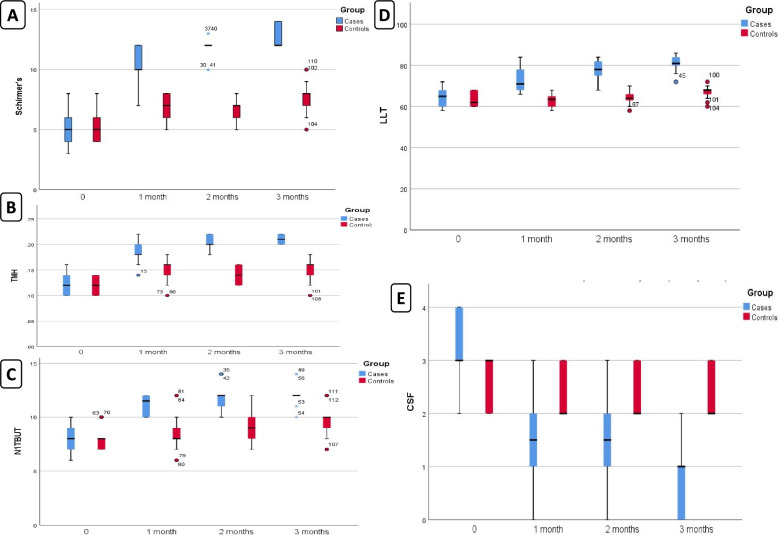
Boxplot showing **A** Schirmer test, **B** Tear meniscus height, **C** Noninvasive tear breakup times, **D** Lipid layer thickness and **E** Corneal fluorescein staining at baseline compared to 1, 2, and 3 months in the injected eye group and control group

Injected eye group showed improvement for Schirmer test (Fig. 1A), TMH (Fig. 1B), NITBUT (Fig. 1C), LLT (Fig. 1D) and CSF (Fig. 1E) at 1 month, 2 months and 3 months when compared to baseline which was statistically significant with *p* < 0.001.

## Discussion

The vital goal of DED treatment is to get homeostasis of the ocular surface and tear film back by breaking the vicious cycle of the disease [17]. A decrease of LG secretion is the main cause of aqueous tear deficient DED. It may become a target for the immune system with signs of inflammation. This can be due to autoimmune diseases (Sjögren’s syndrome), organ transplantation (graft versus host disease), or simply because of aging [21]. In Sjögren syndrome, the tear hyposecretion can be secondary to an autoimmune infiltration in addition to destruction of the lacrimal gland. The use of blood derivatives is an alternative approach which gains interest in regenerative medicine given its potential to stimulate tissue healing. In the present study we objectively assessed the effect of LG injection of PRP on different ocular surface parameters namely, NIBUT, TMH, LLT, Meibography, CFS and Schirmer test throughout the study period and compared to the data of baseline and to the control group. Subjective assessment through OSDI was used to select cases with severe dry eye. Signficant improvement of the tested ocular surface parameters were consistent with previous published studies [14, 15, 20].

The improvement of Schirmer test and TMH values indicate increase of lacrimal volume either due to suppression of the ongoing inflammatory process or stimulation of the injured acinar system. We observed a significant improvement in NIBUT values in 12 patients (85.71%). This improvement suggests an increase in the stability of the lacrimal film that may support increase in the mucin component secreted the LG and/or the conjunctival goblet cells in addition to the increase in the quality of the lipid layer part of the tear film. The latter is supported by the significant increase of the LLT during the follow up period (*P* < 0.001). The better quality of the lipid layer may reflect an improvement in meibomian gland function, however, there was no significant improvement for both groups regarding the meiboscore after 3 months of follow up in both studied groups (*p* > 0.05) suggesting improvement associated with paracrine regulation rather than structural changes.

The values obtained for conjunctival and corneal staining values were decreased. This change was observed from the 30th day onwards and was more evident on 90 days post-treatment (*p* = 0.001), reflecting an improvement in ocular surface health, the significant improvement was noticed after 2 months and continued afterwards. This score is an important indicator of patient functionality and rehabilitation.

The early case series study of Avila published in 2014, four patients with severe lacrimal dysfunction and severe dry eye were treated using PRP, where platelet rich-activated plasma (1 mL) was injected adjacent to the lacrimal gland. All patients showed subjective improvement of symptoms after 3 months follow up [14, 15]. Moreover, in a published conference abstract in 2019 by Abdalrahman et al., 12 patients (10 females and 2 males) have been diagnosed as keratoconjunctivitis sicca. All had lacrimal and subconjunctival injection of PRP [15]. All patients have shown significant improvement in symptoms of ocular discomfort after PRP injection. However, those studies were early case series with small sample sizes and a lot of limitations regarding their methods.

More recently, a study published in 2019 by Avila et al. and included patients with severe DED diagnosed as Sjogren syndrome. Patients received either PRP or hyaluronic acid eye drops and followed up for 90 days. The study showed that PRP improving tear parameters including a reduction in OSDI, corneal staining, the mean Schirmer value, and TBUT at day 90 [20]. This comes in accordance with our results. However, our study has the advantage of using the ocular surface analyzer with non-invasive estimation of the tear film parameters and gave more detailed information about the changes in ocular surface.

Injection represents a common therapeutic approach once regeneration of determinate tissue is needed. It has been used in regenerating several tissues given the activity of several growth factors which can stimulate the proliferation of stem cells [22]. The rational for PRP use is that it contains different components with known pro-regenerative capabilities [23]. During an inflammatory reaction, the LG is infiltrated in massively proliferating immune cells. Acinar cells and duct epithelial cells express autoantigens that attract an attack by immune cells, with subsequent loss of the glandular cells [24]. The functional cell loss leads to reduced secretion of several growth factors, including epidermal growth factor (EGF) which plays a vital role in enhancing normal differentiation of the epithelium and keeping the homeostasis for the ocular surface and the conjunctival epithelium also turns into a non-secretory epithelium through squamous metaplasia, which additionally affect the ocular surface health [25].

The beneficial effect of PRP injection of the LG may be supported by its positive effect on the glandular cells of the liver through a direct effect on hepatocytes, in addition to an indirect antifibrotic effect together with a protective effect against hepatocyte apoptosis [26]. The demand for restoring the function of lacrimal gland has been intensified due to the current advances in stem cell biology and bioengineering technologies and injection of PRP in LGD seems to be a novel proposed treatment in this field.

Despite the scarcity of published data, we postulate a promising role of PRP injection in treating of DED. To the best of our knowledge, this is a unique study in using the ocular surface analyzer in assessing the enrolled patients. However, we encountered some limitations. For instance, the sample size could have been larger and the follow up could have been extended, also the dosing and time interval between the injections should be standardized in larger samples. We recommend more well designed studies to avoid our limitations.

## Conclusion

In conclusion, PRP injection in severe DED induced a positive effect over different ocular surface parameters both for the quantity of the tear lacrimal volume and its quality as well with a better restoration of ocular surface homeostasis and may open new frontiers for treatment and better understanding of the pathophysiology of the DED. This suggests an ongoing, if not increasing role for platelets and derived products in clinical treatments.

## Data Availability

The datasets generated during and analysed during the current study are not available due to privacy/ethical restrictions but are available from the corresponding author on reasonable request.

## Abbreviations

DED: Dry eye disease
AS: Autologous serum
PRP: Platelet rich plasma
OSA: Ocular surface analyzer
NIBUT: Noninvasive tear breakup times
TMH: Tear meniscus height
LLT: Lipid layer thickness
CSF: Corneal fluorescein staining
